# Supplementary figures and images for: Identification and expression analysis of cytokinin metabolic genes IPTs, CYP735A and CKXs in the biofuel plant Jatropha curcas
Source: PeerJ. 2018 May 16;6:e4812. doi: 10.7717/peerj.4812 (PMC5960259; doi:10.7717/peerj.4812)

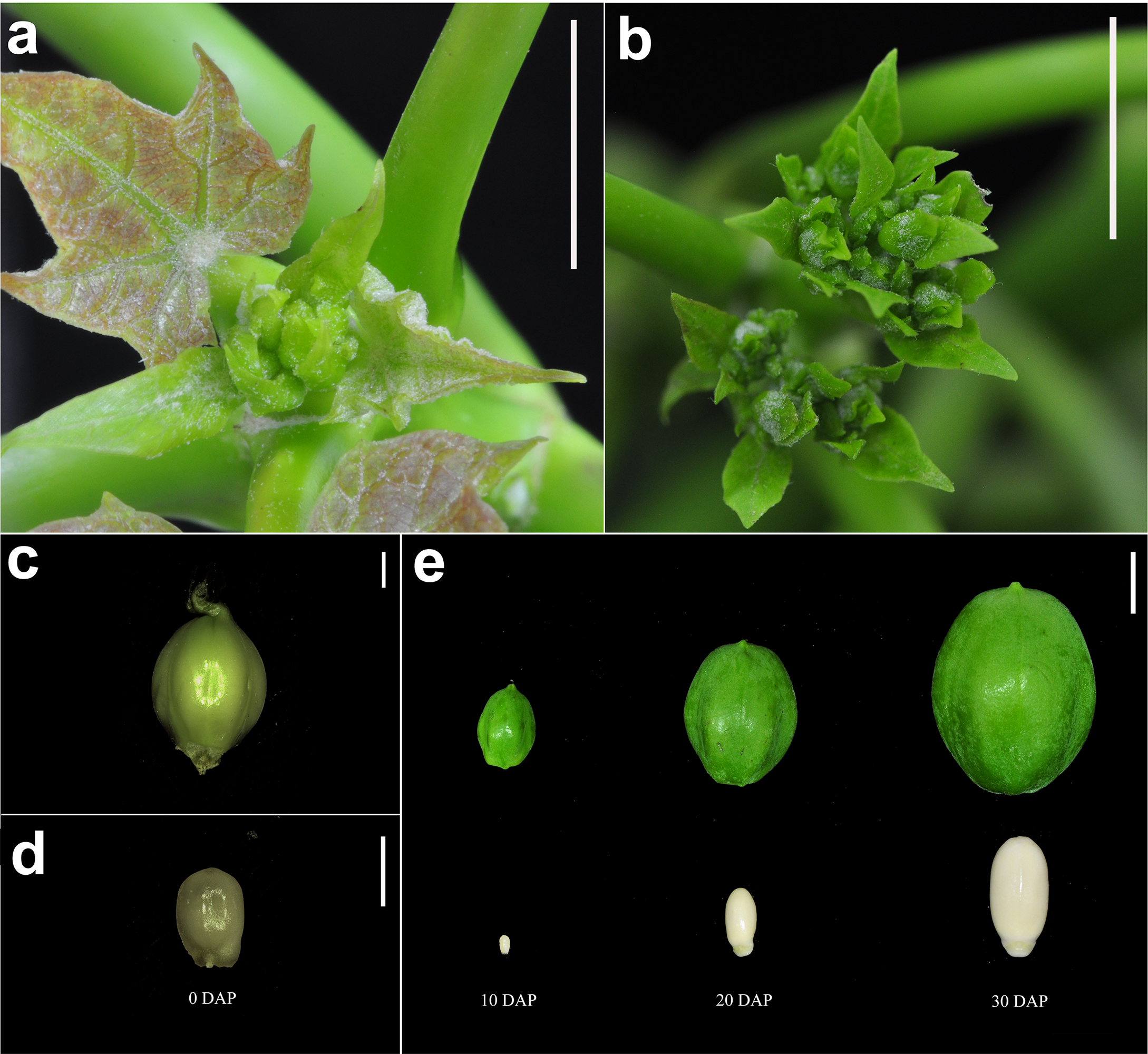

Supplement: Supplemental Information 1 — (A) Flower buds from the FB1 stage. Bar = 1 cm. (B) Flower buds from the FB2 stage. Bar = 1 cm. (C) Unfertilized Jatropha pistil, from which the ovule was obtained. Bar = 1 mm. (D) Morphology of unfertilized ovules. Bar = 1 mm. (E) Morphology of fruits and seeds. The first row contains fruits at different developmental periods. The second row shows the seeds of the corresponding fruits. Bar = 1 cm. 0 DAP, unfertilized ovules; 10 DAP, seeds at 10 days after pollination; 20 DAP, seeds at 20 days after pollination; 30 DAP, seeds at 30 days after pollination; DAP, days after pollination. Photos by Li Cai. [file peerj-06-4812-s001.png]
