# Supplementary material for: Identification and expression analysis of cytokinin metabolic genes IPTs, CYP735A and CKXs in the biofuel plant Jatropha curcas
Source: PeerJ. 2018 May 16;6:e4812. doi: 10.7717/peerj.4812 (PMC5960259; doi:10.7717/peerj.4812)
Supplement: Supplemental Information 2 — Raw data. [file peerj-06-4812-s002.docx]

**Table S1.** GenBank Accession Numbers of the gene sequences in this work.

| Genes | Accession Numbers | Genes | Accession Numbers |
| --- | --- | --- | --- |
| *AtIPT1* | NM_105517.2 | *AtCKX5* | NM_106199.5 |
| *AtIPT2* | NM_128335.2 | *AtCKX6* | NM_116209.3 |
| *AtIPT3* | NM_116176.3 | *AtCKX7* | NM_180532.3 |
| *AtIPT4* | NM_118598.1 | *JcCKX1* | XM_012230841.2 |
| *AtIPT5* | NM_001343584.1 | *JcCKX2* | XM_012232076.2 |
| *AtIPT6* | NM_102352.1 | *JcCKX3* | XM_012221553.2 |
| *AtIPT7* | NM_113267.3 | *JcCKX4* | XM_012232009.2 |
| *AtIPT8* | NM_112803.1 | *JcCKX5* | XM_012216552.2 |
| *AtIPT9* | NM_122011.4 | *JcCKX6* | XM_012213203.2 |
| *JcIPT1* | XM_012226712.2 | *JcCKX7* | XM_012221810.2 |
| *JcIPT2* | XM_012220474.2 | *RcCKX1* | XM_002534412.2 |
| *JcIPT3* | XM_020685440.1 | *RcCKX2* | XM_002513353.2 |
| *JcIPT5* | XM_012214128.2 | *RcCKX3* | XM_002514073.2 |
| *JcIPT6* | XM_012214198.2 | *RcCKX5* | XM_002510404.2 |
| *JcIPT9* | XM_012223167.2 | *RcCKX6* | XM_002513072.2 |
| *RcIPT1* | XM_015724072.1 | *RcCKX7* | XM_002516087.2 |
| *RcIPT2* | XM_002529038.2 | *OsCKX1* | XM_015780365.1 |
| *RcIPT3* | XM_002533018.2 | *OsCKX2* | XM_015773930.1 |
| *RcIPT5* | XM_002517104.2 | *OsCKX3* | XM_015757556.1 |
| *RcIPT6* | XM_002519053.2 | *OsCKX4* | XM_015765857.1 |
| *RcIPT9* | XM_002524218.2 | *OsCKX5* | XM_015770438.1 |
| *OsIPT1* | AB239797.1 | *OsCKX6* | XM_015769432.1 |
| *OsIPT2* | AB239798.1 | *OsCKX7* | XM_015768875.1 |
| *OsIPT3* | AB239799.1 | *OsCKX8* | XM_015778030.1 |
| *OsIPT4* | AB239800.1 | *OsCKX9* | XM_015783650.1 |
| *OsIPT5* | AB239801.1 | *OsCKX10* | XM_015786878.1 |
| *OsIPT6* | AB239803.1 | *OsCKX11* | XM_015795175.1 |
| *OsIPT7* | AB239804.1 | *AtCYP735A1* | NM_123206.3 |
| *OsIPT8* | AB239805.1 | *AtCYP735A2* | NM_105381.5 |
| *OsIPT9* | AB239806.1 | *JcCYP735A* | XM_012222581.2 |
| *AtCKX1* | NM_001336920.1 | *RcCYP735A* | XM_002516677.2 |
| *AtCKX2* | NM_127508.2 | *OsCYP735A3* | XM_015794046.1 |
| *AtCKX3* | NM_125079.3 | *OsCYP735A4* | XM_015755276.1 |
| *AtCKX4* | NM_119120.2 |  |  |
