# Supplementary material for: Identification and expression analysis of cytokinin metabolic genes IPTs, CYP735A and CKXs in the biofuel plant Jatropha curcas
Source: PeerJ. 2018 May 16;6:e4812. doi: 10.7717/peerj.4812 (PMC5960259; doi:10.7717/peerj.4812)
Supplement: Supplemental Information 3 [file peerj-06-4812-s003.docx]

**Table S2** Sequences of PCR primers used in this work (F, forward; R, reverse)

| **Genes** |  | | **Sequences(5’-3’)** |
| --- | --- | --- | --- |
| *JcIPT1* | F | CTTATTCTCATGTAGAGGACAG | |
|  | R | GCACCAAACAACTTTGTTG | |
| *JcIPT2* | F | CAAGAGTCACAGATGGAGAG | |
|  | R | GCACAATCGTCACATAAGC | |
| *JcIPT3* | F | TGTCTATTACTTCTCTTTCCCA | |
|  | R | ATTTTCGACAAATATGGATGA | |
| *JcIPT5* | F | TGCTTCCTTATCATCCATCT | |
|  | R | ACCAACTCCTATCAATCCTT | |
| *JcIPT6* | F | GTGGGAGGCTCAAACAACTACA | |
|  | R | ACCTGATTGCACCATAAGATCG | |
| *JcIPT9* | F | GATAATGAGCGAAGTATGTG | |
|  | R | TTGGTGAGCCGTAGTTAA | |
| *JcCKX1* | F | GCATAAGCTCCTCTGCACCTC | |
|  | R | CTGCTTGTGGAAACTCAACGA | |
| *JcCKX2* | F | TCTCCACTCTTAACAACAAACA | |
|  | R | TTAAATGAATTAATTTAAATGAATA | |
| *JcCKX3* | F | ATCACCAGTCACCACTACCA | |
|  | R | CTCTATGATCATTTTCATACACCTG | |
| *JcCKX4* | F | ATATACACCAGCACCCCCTTC | |
|  | R | ACCTCAACTTAACGGTCATCA | |
| *JcCKX5* | F | CCAACAAGGAGCAGAACCGA | |
|  | R | GACTGGCGAGCTGTGTATGT | |
| *JcCKX6* | F | AGTGACCGTGGGACTCTCTT | |
|  | R | ACTAGCTCCTGCAGCTTTCC | |
| *JcCKX7* | F | CACCGGATAATTTGACTTGC | |
|  | R | AGTATTCCTTTACATTGCTTCA | |
| *JcCYP735A* | F | AACTCCCTCTTCTTACCCCAG | |
|  | R | TATCTTTTCATGGATTTAAGGGCT | |
