# Supplementary material for: Identification and expression analysis of cytokinin metabolic genes IPTs, CYP735A and CKXs in the biofuel plant Jatropha curcas
Source: PeerJ. 2018 May 16;6:e4812. doi: 10.7717/peerj.4812 (PMC5960259; doi:10.7717/peerj.4812)
Supplement: Supplemental Information 4 [file peerj-06-4812-s004.docx]

**Table S3** Sequences of qRT-PCR primers used in this work (F, forward; R, reverse)

| **Genes** |  | | **Sequences(5’-3’)** |
| --- | --- | --- | --- |
| *JcIPT1* | F | TCAGTTCCACTCCCATTACATTT | |
|  | R | TGATTTACCACACCCAGTTGCTC | |
| *JcIPT2* | F | ATTCAGGCTCTGGTGAGTCCATTCC | |
|  | R | TTCCACCATACTCAGGCGTCTCA | |
| *JcIPT3* | F | CAGAGTTCTTCAGGAAGCAATACGC | |
|  | R | GGCTGTCAAACTGTAAAGAAACCG | |
| *JcIPT5* | F | GGCATCAAGCCTCGGACTCTG | |
|  | R | AGGCAATGACACATCCACCCA | |
| *JcIPT6* | F | GTGGGAGGCTCAAACAACTACA | |
|  | R | ACCTGATTGCACCATAAGATCG | |
| *JcIPT9* | F | CCAATACCACCTCCTCGCCAC | |
|  | R | GACCCAACATCAAGACCACGG | |
| *JcCKX1* | F | AAGGTCAAGCACAGGCTCATCA | |
|  | R | CCAATGGTGAGGTGAAGGTAATCT | |
| *JcCKX2* | F | GGCTTACAACGGAGTGGTAGTGG | |
|  | R | CATCAATCCATAGTTGCTCTCCTCC | |
| *JcCKX3* | F | TGTCCCTATTTATCCAACATCGCA | |
|  | R | AAAAACTCAACATATCCCACATCTTTCT | |
| *JcCKX4* | F | ATGAAATCAGTTACTTGGAAGGTGGAA | |
|  | R | GCAGATGATCCTATGGTGGTGGC | |
| *JcCKX5* | F | CGTCGTTACAGGCAAAGGTGA | |
|  | R | GGTAGATGGGTTTCCGTAGAGG | |
| *JcCKX6* | F | CTGGAGGCGAGTTGTGGAT | |
|  | R | TGCTTGCCCACTAATCCCT | |
| *JcCKX7* | F | CAAGCCTTCATTTACGGACCACA | |
|  | R | TGATGCCAAACTGACCGAGACC | |
| *JcCYP735A* | F | GAACCTCGCCCTCTTACCG | |
|  | R | GGCAAGAGGCGATCAACTATG | |
| *JcACTIN2* | F | CTCCTCTCAACCCCAAAGCCAA | |
|  | R | CACCAGAATCCAGCACGATACCA | |
